# Acupuncture Can Regulate the Distribution of Lymphocyte Subsets and the Levels of Inflammatory Cytokines in Patients With Mild to Moderate Vascular Dementia

**DOI:** 10.3389/fnagi.2021.747673

**Published:** 2021-11-29

**Authors:** Hui Zhi, Yao Wang, Shichen Chang, Pan Pan, Zhenzhen Ling, Zhen Zhang, Zhinan Ma, Runmin Wang, Xuezhu Zhang

**Affiliations:** ^1^First Teaching Hospital of Tianjin University of Traditional Chinese Medicine, Tianjin, China; ^2^National Clinical Research Center for Chinese Medicine Acupuncture and Moxibustion, Tianjin, China; ^3^Second Clinical Medical College, Yunnan University of Traditional Chinese Medicine, Kunming, China; ^4^Department of Immune Regulation, Immunology Frontier Research Center, Osaka University, Osaka, Japan; ^5^Weifang Traditional Chinese Hospital, Weifang, China

**Keywords:** vascular dementia, “sanjiao” acupuncture method, peripheral immune function, FCM, ELISA

## Abstract

**Background:** Vascular dementia (VD) is the second most common type of dementia after Alzheimer’s disease, but there is a lack of definitive treatment for VD. Acupuncture treatment is effective in improving the cognitive impairment and behavioral capacity of patients with VD. In recent years, more studies indicated that peripheral inflammation and abnormal peripheral immune function may aggravate neuroinflammation and cognitive dysfunction. However, there are few studies about the acupuncture and the abnormal peripheral immune function of VD. Also, few studies concern the regulating effect of acupuncture on peripheral immunity of patients with VD.

**Objective:** The aim of this study was to explore the effect of the “sanjiao” acupuncture method on peripheral immunity of patients with mild to moderate VD.

**Methods:** A total of 30 patients with VD were involved in the acupuncture group (AG), which was treated with the “sanjiao” acupuncture method once a day for six times a week and lasted for 12 weeks, and 30 healthy elderly people were assigned to the normal group (NG), which had no treatment. The distribution of lymphocyte subsets and the levels of some inflammatory cytokines in the peripheral blood of subjects were evaluated using the flow cytometry (FCM) and the enzyme-linked immunosorbent assay (ELISA).

**Results:** A total of 60 subjects were involved in this study, while 58 subjects completed the entire trial. Before treatment, the levels of CD3^+^ T, CD4^+^ T cells, CD4^+^/CD8^+^, Tregs, B cells, IFN-γ, and IL-10 in patients with VD were significantly decreased compared with the normal group (all *P* < 0.05 or *P* < 0.01). The level of TNF-α in peripheral blood of patients with VD was significantly increased (*P* < 0.01). After acupuncture treatment, the levels of CD3^+^ T, CD4^+^ T cells, and IFN-γ were significantly increased (all *P* < 0.05 or *P* < 0.01). The level of TNF-α was significantly decreased (*P* < 0.01). The proportion of Tregs was increased (*P* < 0.01), but it was still lower than that of the normal group (*P* < 0.05).

**Conclusion:** The acupuncture method can increase the proportion of CD3^+^, CD4^+^ T cells, and Tregs in peripheral blood of patients with VD. And, it reduces the levels of pro-inflammatory factor TNF-α, which achieves the anti-inflammatory effects and immunostimulation. It suggests that acupuncture can improve the peripheral immune dysfunction of patients with VD by regulating the distribution of lymphocyte subsets and the levels of inflammatory cytokines.

**Clinical Trial Registration:** [www.chictr.org.cn], identifier [ChiCTR-IOR-17012052].

## Introduction

Vascular dementia (VD) is the general term of dementia syndrome caused by the injury of brain tissue resulting from a series of cerebral vascular diseases. Its clinical features are mainly cognitive dysfunction and neurological dysfunction, which are related to the cerebrovascular diseases. VD is the second most common type of dementia after Alzheimer’s disease (AD), accounting for about 20% of all dementia populations (O’Brien and Thomas, 2015; Dichgans and Leys, 2017).

During the progress of VD, chronic cerebral hypoperfusion (CCH) and thromboembolism lead to a decrease in cerebral blood flow, causing anoxia, oxidative stress, and inflammatory response and finally leading to a cognitive defect (Venkat et al., 2015). Neuroinflammation plays a key role in the occurrence and progress of VD (Iadecola, 2010). After cerebral ischemia, the microglial cells are activated and release the pro-inflammatory factors, chemokines, and reactive oxygen species, which can damage the neuron. Meanwhile, these inflammatory mediators further promote the activation of microglial cells, which aggravate the inflammatory reaction. Otherwise, brain tissue releases high mobility group protein B1 (HMGB1), hypoxia-inducible factor-1α (HIF-1α), brain-derived antigen, and other signals after ischemia which activate the peripheral immune system through the purinoceptors, Toll-like receptors, and receptors for activated glycation end products. Then, the lymphocyte subsets transform into the pro-inflammatory phenotype. Monocytes and macrophages are activated, and the pro-inflammatory factors are increased. All of these affect the immune function of patients with VD. Moreover, the immune cells such as neutrophils, monocyte, and macrophages in the peripheral blood and the pro-inflammatory factors that they released invade into brain parenchyma *via* damaged blood-brain barrier (BBB), further promoting the activation of microglial cells and aggravating the neuroinflammation which exacerbate the nerve injury.

Previously, the effect of inflammation of the central system on cognitive function is of great concern for a long time. However, in recent years, more studies indicate that peripheral inflammation and abnormal peripheral immune function may aggravate neuroinflammation and neuron damage (An et al., 2014; Prinz and Priller, 2017). More attention has been paid to the promotion of peripheral inflammation on the neuroinflammation, and the important role of the peripheral immune cells, especially T cells, in maintaining normal cognitive function.

For example, intraperitoneal injection of lipopolysaccharide (LPS), which induced peripheral inflammation, can cause behavioral changes and cognitive impairment in mice. Meanwhile, in the LPS-induced mice, the microglial cells are activated, neuronal cells are decreased, the NF-kB signaling pathway is activated, the expression of pro-inflammatory cytokines (TNF-α and IL-1β) is increased, and the expression of anti-inflammatory cytokines (IL-4 and IL-10) is decreased (Zhao et al., 2019). The LPS cannot cross the blood-brain barrier (BBB), but the intraperitoneal injection of LPS can cause cognitive impairment. The peripheral inflammation may result in damages in the BBB, and peripheral immune cells infiltrate the brain parenchyma, causing neuroinflammation. The rats with hyperammonemia show a rapid and reversible induction of peripheral inflammation, with activation of astrocyte and microglial cells. Meanwhile, the expression of TNF-α, IL-1β, and gamma-aminobutyric acid (GABA) is increased, and the abilities of motor coordination and learning are impaired. These abnormal conditions can be prevented using peripheral anti-TNF-α treatment (Balzano et al., 2020).

T cells are the core of cellular immune response. When the body loses T cells, the B cells, mast cells, macrophages, and dendritic cells would transform into the pro-inflammatory phenotype, activate the immune system, release the pro-inflammatory factors into the brain and peripheral blood, and then inhibit the brain function. The cognitive function of rats with a shortage of T cells in peripheral blood is impaired, but the cognitive impairment can be improved after T-cell transplantation. However, the cognitive impairment induced by drugs and the abnormal behaviors can also have a recovery after T-cell transplantation (Radjavi et al., 2014). Research shows that the inhibition of CD4^+^ T cells results in hippocampus neuron damage and cognitive impairment (De Miranda et al., 2017). These data indicate that peripheral inflammation and immunologic injury can cause and aggravate neuroinflammation, and result in cognitive impairment and many other changes. The T cells, especially CD4^+^ T cells, play a key role in maintaining the normal cognitive function. Otherwise, it is proved that the immune response plays an important role in cerebral small-vessel disease and VD (Raz et al., 2015). Many studies show that there are peripheral inflammation and abnormal peripheral immune function in VD, which include increased pro-inflammatory factors (IL-1β, IL-6, TNF-α, IFN-γ), decreased T cells, the imbalance between the Th17 and Treg, and so on (Dadsetan et al., 2016; Balzano et al., 2019).

Professor Han Jingxian summed up his many years of clinical experience and created the “sanjiao” acupuncture method to treat VD and achieved good clinical results. Our previous studies had shown that the “sanjiao” acupuncture method could significantly improve the cognitive impairment and behavioral capacity of patients with VD (Yu et al., 2006). Some studies indicate that the acupuncture treatment has anti-inflammatory and immunoregulation effects, and it is effective on VD (Shi et al., 2012; Chen et al., 2015, 2017; Si et al., 2016). Furthermore, acupuncture can improve the mitochondrial function of hippocampus and protect the nerves by the way of antioxidation, regulating cell proliferation, anti-apoptosis, etc. (Wang et al., 2009; Zhang et al., 2014).

At present, there are few studies about acupuncture and abnormal peripheral immune function of VD. And, few studies concern the regulating effect of acupuncture on the peripheral immunity of patients with VD. There is a close connection between the peripheral immune and the central nervous system, and regulating the peripheral immunity can influence the central nervous system and cognitive function. So, we think that regulating the peripheral immunity may be beneficial to the cognitive function of patients with VD. Therefore, this study investigates the regulatory effect of acupuncture treatment on peripheral immune function of patients with VD.

## Materials and Methods

### Trial Design

**FIGURE 1:**
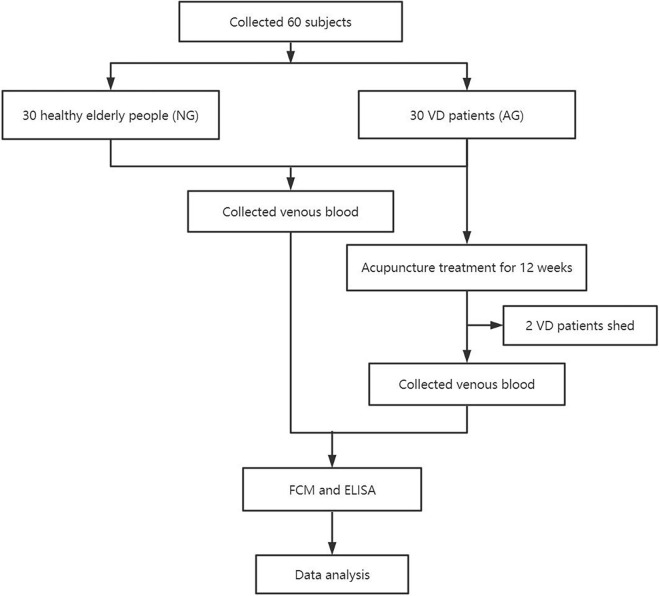
Research flowchart.

This trial involved 30 patients with VD as the acupuncture group (AG), and 30 healthy elderly people were assigned to the normal group (NG). And, the trial lasted for 12 weeks. The distribution of lymphocyte subsets and the levels of some inflammatory cytokines in the peripheral blood of patients with VD before and after treatment were evaluated using FCM and ELISA. The distribution of lymphocyte subsets and the levels of some inflammatory cytokines in the peripheral blood of the healthy elderly people were also evaluated at the beginning of the trial. The study was performed according to the common guidelines for clinical trials (Declaration of Helsinki, International Conference on Harmonization/WHO Good Clinical Practice standards including certification by an external audit). The trial protocol was approved by the Research Ethical Committee of First Teaching Hospital of Tianjin University of Traditional Chinese Medicine. The research flow chart is shown in Figure 1.

### Participants

This study included 30 patients with VD from the First Teaching Hospital of Tianjin University of Traditional Chinese Medicine. The inclusion criteria for VD referred to the National Institute for Neurological Disorders and Stroke (NINDS/AIREN) in 1993 and the *Diagnostic and Statistical Manual of Mental Disorders (DSM-5)*. And, the determination of mild to moderate dementia was accorded to the Clinical Dementia Rating Table (CDR) criteria. Patients were also required to have a Mini-Mental State Examination (MMSE) score ≤ 24 (above secondary school); ≤ 20 (primary school level); ≤ 17 (illiteracy), Hachinski Ischemic Scale score ≥ 7, and Hamilton Depression Scale score ≤ 17. Otherwise, patients who enrolled in this trial should be aged 55–80 years, and the female should be post-menopausal women with no fertility (at least 1 year after menopause). The principal exclusion criteria included the presence of other types of dementia or nerve and cerebral diseases (brain tumor, Parkinson’s disease, multiple sclerosis, amyotrophy, myasthenia gravis, depressive syndrome, and severe neurological deficits). Patients suffering from severe heart, lung, liver, kidney, hematopoietic system, endocrine system diseases, and other diseases that can affect cognitive function were also excluded.

A total of 30 healthy elderly people whose gender and age matching with the AG were involved from the Physical Examination Center of the First Teaching Hospital of Tianjin University of Traditional Chinese Medicine. The exclusion criteria for healthy elderly people included cerebrovascular disease, dementia or other nervous system diseases, malignant tumor, severe hepatic, and renal dysfunction, hematopathy, HIV infection, recent bacterial or viral infection, chronic organic diseases, immunodeficiency disease, contact history of chemical poisons, and the personal history of current use of medications that can affect the immune system. Healthy elderly people who had hypertension, diabetes, coronary heart disease, and other internal medicine diseases that did not control stably and people who had psychogenia such as anxiety and depression were also excluded. All the participants signed the “informed consent form” and consented to undergo the relevant examination.

### Interventions

In the AG, the patients with VD received the “sanjiao” acupuncture method, which included Danzhong (CV17), Zhongwan (CV12), Qihai (CV6), bilateral Xuehai (SP10), Zusanli (ST36), and Waiguan (SJ5). One-off sterile acupuncture needles (Huatuo, Suzhou Medical Instruments Factory, Suzhou, China), with a length of 40 mm and diameter of 0.25 mm, were used in the trial. Needles were inserted horizontally 15 mm into CV17 and SJ5; 25–40 mm perpendicularly into CV12, CV6, and ST36; 15–25 mm obliquely into SP10. After getting the needle sensation, the needles were retained *in situ* for 20 min. The patients were given the acupuncture treatment once a day, 6 times a week for 12 weeks. And, the acupuncture therapy was performed by professional acupuncturists. The normal group had no interventions.

### Sample Collection and Evaluation

At the beginning of the study, 4 ml venous blood of every healthy individual in the normal group was collected in the EDTA tube for FCM and ELISA in the morning. Out of 4 ml, 2 ml venous blood sample was anticoagulated using EDTA-K2. Then, the blood was transferred into the centrifuge tube. The 3 × Red Blood Cell Lysis Buffer (Beijing Solarbio Science and Technology Co., Ltd., Beijing, China) was added into the tube and mixed thoroughly. Then, it was incubated at 4°C for 15 min in the dark (reverse mixing two times during the period). And, it was centrifuged at 450 × *g* for 10 min to precipitate leukocytes. Later, the supernate was abandoned and the 2 × Red Blood Cell Lysis Buffer was added into the leukocyte precipitation. It was mixed thoroughly and incubated at 4°C for 10 min in the dark (reverse mixing once during the period). Then, it was centrifuged at 450 × *g* for 10 min and the supernate was abandoned. Finally, PBS was added into the tube to resuspend the cells which were then observed under the microscope. The cell concentration was adjusted to 10^6^–10^7^ cells/ml for cell staining. Then, according to the kit instructions, the cells were stained with different biomarker antibodies (Becton, Dickinson and Company, United States; eBioscience, United States). FACS Calibur (BD Biosciences, United States) was used to obtain data and Flow Jo 7.6.1 software (BD Biosciences, United States) was used for analysis.

**FIGURE 2:**
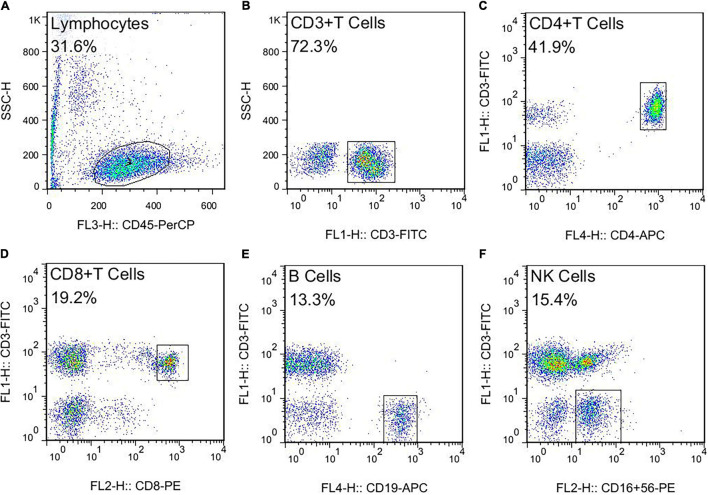
The flow cytometry gating strategy for TBNK. **(A)** Lymphocytes. **(B)** T lymphocytes (CD3^+^ T cells). **(C)** The proportion of CD4^+^ T cells (CD3^+^CD4^+^ T cells). **(D)** The proportion of CD8^+^ T cells (CD3^+^ CD8^+^ T cells). **(E)** The B cells (CD3–CD19^+^ B cells). **(F)** The NK cells (CD3–CD16^+^ CD56^+^ NK cells).

**FIGURE 3:**
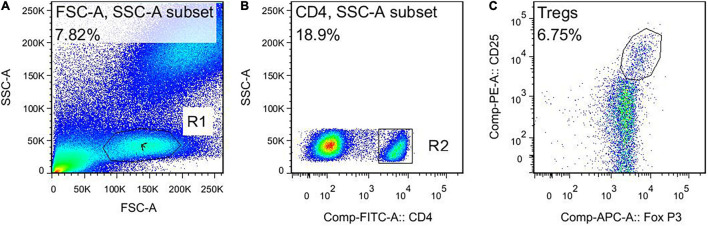
The flow cytometry gating strategy for Treg. **(A)** Lymphocytes. **(B)** The CD4^+^ cells. **(C)** The Treg cells (CD4^+^ CD25^+^ Foxp3^+^ cells).

This study used negative control for flow cytometry (FCM). The FCM gating strategy for TBNK is as follows: this study used four color schemes to analyze the lymphocyte subsets. When the cells were detected, the sample was divided into A tube and B tube. The A tube detected the PerCP-CD45, FITC-CD3, APC-CD4, and PE-CD8, and the B tube detected the PerCP-CD45, FITC-CD3, APC-CD19, and PE-CD16^+^56. The specific gating strategy is shown in Figure 2. First, the lymphocyte gate was set up and named as R1 gate. Then, the lymphocytes (CD45^+^ cells) were confirmed by CD45/SSC, as shown in Figure 2A. Second, the cells were analyzed in the R1 gate. The T lymphocytes (CD3^+^ cells) were confirmed by CD3/SSC, as shown in Figure 2B. The proportion of CD4^+^ T cells (CD3^+^/CD4^+^ cells) were determined by CD3/CD4, as shown in Figure 2C. The proportion of CD8^+^ T cells (CD3^+^/CD8^+^ cells) were determined by CD3/CD8, as shown in Figure 2D. Regarding the B tube, the first and second steps for the gating strategy were the same as for the A tube. Then, the B cells (CD3^–^/CD19^+^ cells) were confirmed by CD3/CD19, as shown in Figure 2E. The NK cells (CD3^–^/CD16^+^56^+^) were determined by CD3/CD16^+^56, as shown in Figure 2F. The flow cytometry gating strategy for Treg is shown in Figure 3. First, the lymphocytes were confirmed by FSC/SSC and named R1 gate, as shown in Figure 3A. Second, the cells were analyzed in the R1 gate. The CD4^+^ cells were determined by CD4/SSC and named as R2 gate, as shown in Figure 3B. Finally, the cells were analyzed in the R2 gate. The Treg cells (CD25^+^/Foxp3^+^ cells) were confirmed by Foxp3/CD25, as shown in Figure 3C.

Another 2 ml venous blood was centrifuged at 4°C 1,500 × *g* for 10 min to separate serum, and then the different cytokine levels in serum were detected using ELISA kits (IBL International GmbH, Hamburg, Germany). The limits of detection were as follows: Human IL-1β 0.3 pg/ml, Human IL-2 9.1 pg/ml, Human IL-4 1.3 pg/ml, Human IL-10 1.0 pg/ml, Human TNF-α 5.0 pg/ml, and Human IFN-γ 0.99 pg/ml. Also, each sample was repeated three times in the test. In the AG, the blood of patients with VD was treated in the same way for testing before and after treatment.

### Statistical Analysis

All statistical analysis was performed using SPSS 21.0 (IBM Corporation, United States). Measurement data with normal distribution were expressed as mean ± standard deviation ($\bar{x}$ ± s), and measurement data with non-normal distribution were expressed as the median and interquartile range (M, Q). Measurement data for normal distribution between the two groups were tested using two independent samples, and measurement data for non-normal distribution were tested using a non-parametric test. The comparison of the conditions of patients before and after treatment was tested using paired sample *t*-test. Chi-square test was used for the comparison of count data, and a non-parametric test was used for the comparison of rank data. The significant difference level was α = 0.05. *P* < 0.05 was considered statistically significant.

## Results

### Comparison of the General Conditions Before Treatment

A total of 60 subjects were involved in this study, while 58 subjects completed the entire trial. Among them, the normal group included 30 healthy people, and the AG included 28 patients with VD. The baseline comparison of subjects of the two groups is shown in Table 1. It showed that among the different groups, there were no significant differences in gender, age, education level, etc. Also, they were comparable.

**TABLE 1:** Comparison of general conditions of the two groups.

	**Normal group (*n* = 30)**	**Acupuncture group (*n* = 28)**	**Aggregate**	**Statistic**	***P*-value**
**Gender: Number (%)**					
Male	13 (43.33)	16 (57.14)	29 (50)	*X*^2^ = 1.105	0.293
Female	17 (56.67)	12 (42.86)	29 (50)		
Age ($\bar{x}$ ± s)	68.53 ± 6.68	68.14 ± 6.90		*t* = –0.219	0.828
**Education level: Number (%)**					
Illiterate (uneducated)	1 (3.33)	2 (7.14)	3 (5.17)	*Z* = –0.314	0.754
Primary school (education period ≤ 6 years)	6 (20)	4 (14.29)	10 (17.24)		
Secondary school (6 years < education period ≤ 12 years)	20 (66.67)	18 (64.29)	38 (65.52)		
University (education period > 12 years)	3 (10)	4 (14.29)	7 (12.07)		

### Percentage of the Lymphocyte Subsets in Peripheral Blood

Before treatment, the ratio of CD3^+^, CD4^+^ T cells, Tregs, B cells to total lymphocytes and CD4^+^/CD8^+^ ratio of patients with VD were significantly lower compared with the normal group (all *P* < 0.05).

After the acupuncture treatment, the proportion of CD3^+^, CD4^+^ T cells, and Tregs in the peripheral blood of patients with VD in the AG was significantly increased compared with that before treatment (all *P* < 0.05). There was no significant change in the proportion of CD8^+^ T cells, CD4^+^/CD8^+^, B cells, and NK cells after treatment in the AG (all *P* > 0.05).

**FIGURE 4:**
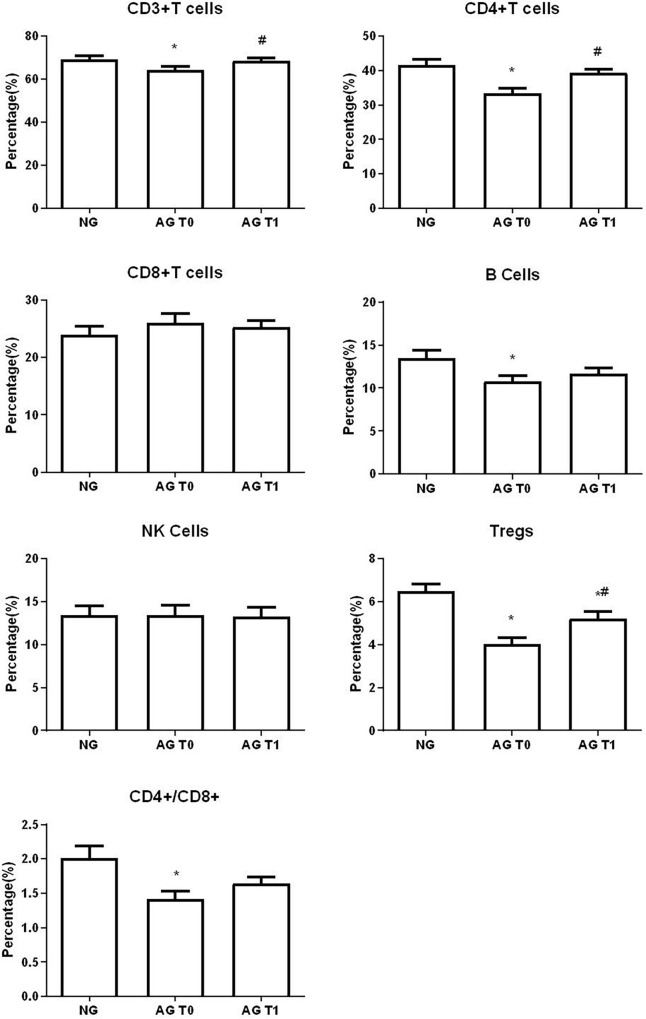
Percentage of lymphocyte subsets in peripheral blood. NG, normal group; AG, acupuncture group; T0, before treatment; T1, after treatment. *Compare with the healthy elderly people, *P* < 0.05. ^#^Compare with the conditions before treatment, *P* < 0.05.

**FIGURE 5:**
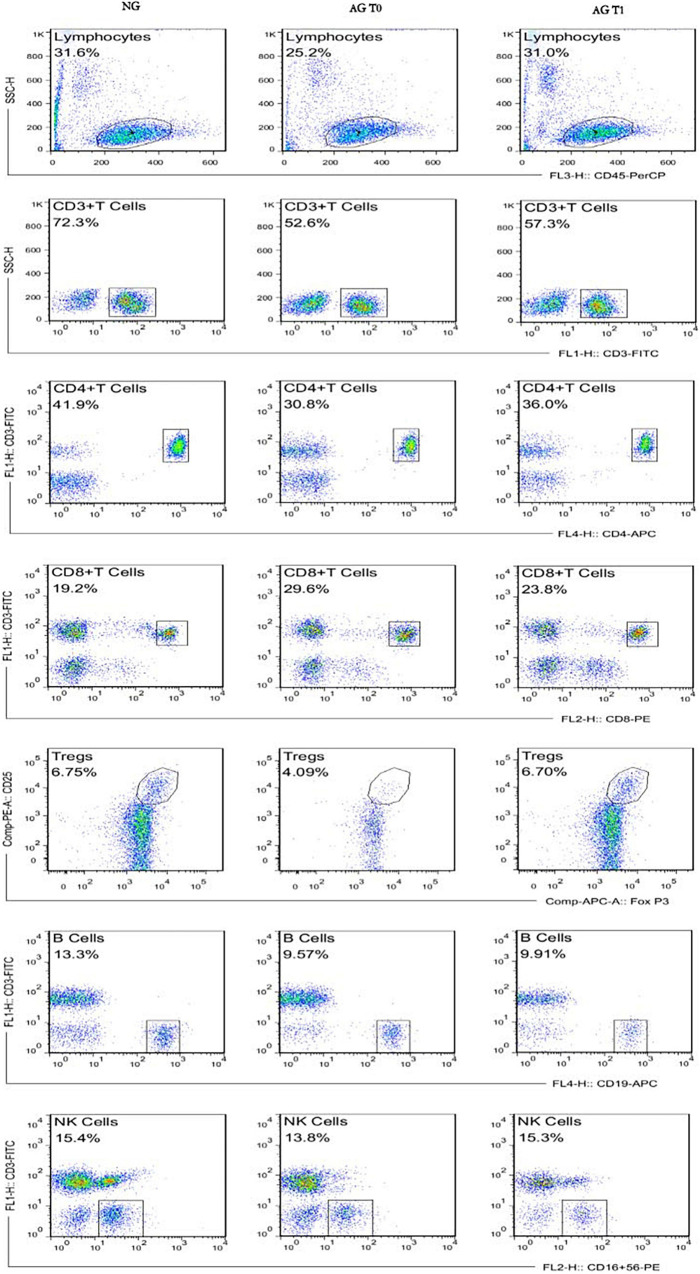
Comparison of lymphocyte subsets in peripheral blood of different groups.

In contrast, after the acupuncture treatment, the proportion of Tregs in patients with VD was still low compared with the normal group (*P* < 0.05). And after the treatment, the proportion of CD3^+^, CD4^+^ T cells, B cells, and the ratio of CD4^+^/CD8^+^ were slightly lower compared with the normal group, but there was no statistical difference between the two groups (all *P* > 0.05). The proportion of CD8^+^ T cells was slightly higher, but there was no statistical difference between the two groups (*P* > 0.05). There was no significant difference in the proportion of NK cells compared with the normal group after treatment (*P* > 0.05) (Figures 4, 5).

### Cytokine Levels in Peripheral Blood

Before treatment, the levels of IL-10 and IFN-γ in the peripheral blood of patients with VD were significantly reduced compared with the normal group (*P* < 0.05). The content of TNF-α was increased significantly (*P* < 0.05). There were no significant changes in IL-1β, IL-2, and IL-4 between the two groups (*P* > 0.05).

After acupuncture treatment, the levels of IL-4 and TNF-α in the peripheral blood of patients with VD were significantly reduced in the AG compared with that before treatment (*P* < 0.05). The IFN-γ level was significantly increased in the AG (*P* < 0.05). The levels of IL-1β and IL-2 of patients with VD showed a downward trend, but there was no statistical significance after treatment (*P* > 0.05). There was no significant change in IL-10 after treatment (*P* > 0.05).

**FIGURE 6:**
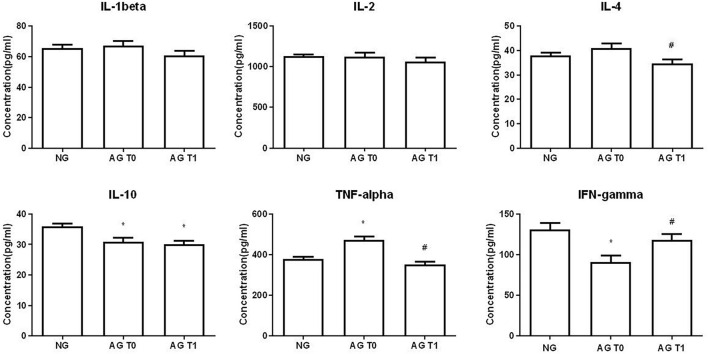
Cytokine concentration in peripheral blood. *Compare with the healthy elderly people, *P* < 0.05. ^#^Compare with the conditions before treatment, *P* < 0.05.

Compared with the healthy elderly people, the level of IL-10 was still lower after acupuncture treatment (*P* < 0.05). The contents of IL-1β, IL-2, IL-4, TNF-α, and IFN-γ were slightly lower, and there was no statistical difference between the two groups (*P* > 0.05) (Figure 6).

## Discussion

Our previous studies had shown that the “sanjiao” acupuncture method could significantly improve the cognitive impairment and behavioral capacity of patients with VD. Also, there were no adverse effects caused by the acupuncture treatment (Yu et al., 2006). Moreover, during the course of this study, the cognitive function of patients with VD was improved after the acupuncture treatment, and they had no significantly related adverse reactions except for the minor bleeding caused by needle acupuncture, which suggested that acupuncture treatment for VD was safe and effective. Hence, this study further investigated the regulatory effect of acupuncture treatment on the peripheral immune function of patients with VD and confirmed that the “sanjiao” acupuncture method could increase the proportion of CD3^+^, CD4^+^ T cells, and Tregs in the peripheral blood of patients with VD. Also, it reduced the levels of pro-inflammatory factor TNF-α, which achieved the anti-inflammatory effects and immunostimulation. This suggested that acupuncture could improve the peripheral immune dysfunction of patients with VD by regulating the distribution of lymphocyte subsets and the level of inflammatory cytokines.

Vascular dementia is the second most common type of dementia after Alzheimer’s disease, and it is caused by a decrease in cerebral blood flow and damage to the neurovascular unit (Sinha et al., 2020). There is no doubt that neuroinflammation plays a critical role in the occurrence and progress of VD (Iadecola, 2010). However, in recent years, the promotion of peripheral inflammation on the neuroinflammation, and the important role of the peripheral immune cells, especially T cells, in maintaining normal cognitive function, have been paid more attention. Evidence indicates that there are peripheral inflammation and abnormal immune function in VD, which are closely associated with cognitive disorder (Schmitz et al., 2015; Busse et al., 2017; Guo et al., 2019). Research shows that lipopolysaccharide (LPS) injection can activate microglial cells and result in an increased production of IL-1β and TNF-α in the rat hippocampus, which finally induces learning and memory deficits (Tanaka et al., 2006). The hippocampus nerve of the immunodeficient mice is significantly impaired, and it cannot be induced by a complex environment. The structure and function of the hippocampal nerve can recover after T cells eliminated the specific central nervous system antigen, which indicates that T cells are the foundation of hippocampal plasticity and cell renewal. Research shows that the CD4^+^ T cells are the most important immune cells for learning. The learning ability of rats is decreased after using anti-CD4, but not after using anti-CD8 (Prinz and Priller, 2017). The T cells, especially CD4^+^ T cells, may be the most important lymphocyte subsets in maintaining normal cognitive function.

### Distribution of Lymphocyte Subsets in the Peripheral Blood of Patients With Vascular Dementia

A study shows that VD has a pronounced impact on the peripheral immunity. The CD3^+^, CD4^+^, and CD8^+^ T cells, B cells, and NK cells are severely decreased in patients with VD (Busse et al., 2017). Our study found that before treatment, the proportion of CD3^+^ and CD4^+^ T cells, and the ratio of CD4^+^/CD8^+^ in patients with VD were significantly lower than those in normal elderly people. This result was similar to the previous studies, which suggested that the peripheral immune function of patients with VD had decreased. And after acupuncture treatment, the proportion of CD3^+^ and CD4^+^ T cells in the peripheral blood was increased, indicating that acupuncture could enhance cellular immune function.

Regulatory T cell (Treg) is a special CD4^+^ T cell subset that can maintain immune homeostasis, protect autoimmunity, and prevent excessive inflammation (Pacella and Piconese, 2019). On the one hand, abnormal Tregs can induce more production of the pro-inflammatory factors (IL-6, IL-17, and IFN-γ), then aggravate the neuroinflammation and cognitive disorder (Van Mierlo et al., 2008). On the other hand, Tregs contribute to the recovery and regeneration of the central nervous system (Zhang et al., 2017; Jiatao et al., 2018). Research indicates that the increased expression of IL-6 and the decline of Tregs may be related to the cognitive disorder of patients with vascular cognitive impairment (VCI) (Guoping et al., 2015). Our study also finds that the percentage of Tregs in the peripheral blood of patients with VD was lower than that of normal elderly people, resulting in the immune inflammation response, thereby affecting the normal immune function of patients with VD. After the acupuncture treatment, the proportion of Tregs increased, indicating that acupuncture treatment could reduce the immune-inflammatory response, reduce the inflammatory damage of tissues and organs, and improve the immune function of patients with VD.

Regarding the B cells and NK cells, the results of our study showed that the proportion of B cells in the peripheral blood of patients with VD was significantly lower than that of normal elderly people, while NK cells were not significantly changed. B cells are mainly involved in humoral immunity, so a decrease in the number of B cells indicates that there is a decline in humoral immunity of patients with VD. However, there was no significant change in the proportion of B cells and NK cells after acupuncture treatment in our study, which may be because the sample size of this study is small, and this part needs further research.

### Cytokines Expression in the Peripheral Blood of Patients With Vascular Dementia

Interleukin is one of the inflammatory cytokines related to VD, which is involved in the development and pathophysiology of VD (Chang et al., 2011). The researchers observed a significantly increased level of IL-1β, TNF-α, IFN-γ, IL-4, and IL-5 (Schmitz et al., 2015). However, the results of our study showed that the levels of IL-1β, IL-2, and IL-4 in the peripheral blood of patients with VD were not significantly different from those of normal elderly people, which may be due to the small sample size of this study and the degree of dementia in this trial was mild compared with other studies. Other related clinical studies are rarely reported, indicating that this part needs further exploration and research.

A study finds that the IL-10 level in the cerebrospinal fluid (CSF) is decreased in VD (Kaiser et al., 2014). Our study also found that the level of IL-10 in the peripheral blood of patients with VD was significantly reduced compared with that of the normal people before treatment. It could be speculated that the decreased expression of IL-10 in the peripheral blood of patients with VD enhanced the inflammatory response, which played a crucial role in the progress of VD.

A study reports that the pro-inflammatory cytokines TNF-α, IL-6, IL-1β, IL-2, and IL-18 in the peripheral blood samples of patients with AD are increased significantly (Lai et al., 2017). Elevated serum TNF-α is confirmed in both patients with VD and VD rat models, and it is closely related to the degree of dementia and prognosis of patients (Sheng et al., 2012; Liu et al., 2014). Studies show that TNF-α is closely related to cognitive function, and the elevation of TNF-α can cause acute cognitive disorder (Hennessy et al., 2017; Magalhães et al., 2018). Otherwise, research shows that the increase of TNF-α level in peripheral blood is related to the decrease of hippocampal volume (Sudheimer et al., 2014), which indicates that the TNF-α level plays a key role in the neurodegeneration in the hippocampus. Our study also found that the TNF-α level in the peripheral blood of patients with VD was increased, which was consistent with the results obtained in previous studies. After the acupuncture treatment, the TNF-α level was decreased, indicating that acupuncture could improve the peripheral inflammation.

IFN-γ is a kind of cytokine, which is released by Th1 cells, cytotoxic T cells, and NK cells (Mosser and Edwards, 2008; Colton, 2009). Some studies discover that the increased expression levels of IL-6 and IFN-γ are bound up with deteriorating cognitive deficits in patients with VCI (Guoping et al., 2015). But some other studies have shown that patients with stroke have a rapid decrease in T lymphocytes and a persistent decrease in INF-γ level (Klehmet et al., 2009). Otherwise, under the pathological conditions, IFN-γ plays an important role in the activation of microglial cells. It can cause the overreaction of microglial cells, induce the proliferation and activation of microglial cells, then cause the release of pro-inflammatory factors and cytotoxic substances, and result in neuronal death in the end (Colonna and Butovsky, 2017; Prinz and Priller, 2017). Some studies find that the effect of IFN-γ on ischemic stroke may be a double-edged sword. On the one hand, it can cause inflammation after cerebral ischemia and aggravate the damage. On the other hand, it has the neuroprotective and neuroregulation effect to some extent. And it can influence the activation of endogenous nerve cells (Wong et al., 2004; Cossetti et al., 2014; Llorens-Bobadilla et al., 2015; Fantetti et al., 2016). The results of our study suggested that compared with the normal subjects, the IFN-γ level in the peripheral blood of patients with VD was significantly reduced before treatment. After the acupuncture treatment, the IFN-γ level in the peripheral blood of patients with VD was increased, but it was still lower than that of normal people. The clinical research results about the IFN-γ are contradictory and the effect of IFN-γ is unclear, which needs to be further explored.

### “Sanjiao” Acupuncture Method for Vascular Dementia

As a part of traditional Chinese medicine, acupuncture has unique advantages in treating VD (Shi et al., 2012). A study shows that acupuncture can improve the behavior and psychological symptoms of dementia (Harris et al., 2019). Acupuncture plays an integral role in antioxidation, anti-apoptosis effect, improving cerebral blood flow, and mitochondrial dysfunction in VD (Wang et al., 2009; Zhang et al., 2014; Li et al., 2015).

Professor Han Jingxian summed up his many years of clinical experience and created the “sanjiao” acupuncture method. The prescription included “Danzhong” (CV17), “Zhongwan” (CV12), “Qiha” (CV6), bilateral “Xuehai” (SP10), “Zusanli” (ST36), and “waiguan” (SJ5) six acupoints. It could treat a variety of senile diseases and achieve good clinical results. Previous studies have shown that the “sanjiao” acupuncture method can significantly improve cognitive impairment and behavioral capacity in patients with VD (Yu et al., 2006).

On the one hand, this study further confirmed that the “sanjiao” acupuncture method could increase the proportion of CD3^+^, CD4^+^T cells, and Tregs in the peripheral blood of patients with VD. On the other hand, it reduced the levels of pro-inflammatory factor TNF-α, which achieved the anti-inflammatory effects and immunostimulation. Anti-inflammatory treatment could reduce the inflammatory damage of tissues and organs, and it could also improve immune function. Immunostimulation reduced the inflammatory response by improving immune function. Therefore, acupuncture achieved the effect of improving peripheral immune function in patients with VD.

## Conclusion

The acupuncture method can increase the proportion of CD3^+^, CD4^+^T cells, and Tregs in the peripheral blood of patients with VD. Also, it reduces the levels of pro-inflammatory factor TNF-α which achieves the anti-inflammatory effects and immunostimulation. It is suggests that acupuncture can improve the peripheral immune dysfunction of patients with VD by regulating the distribution of lymphocyte subsets and the levels of inflammatory cytokines.

## Data Availability Statement

The raw data supporting the conclusions of this article will be made available by the authors, without undue reservation.

## Ethics Statement

The studies involving human participants were reviewed and approved by the Research Ethical Committee of First Teaching Hospital of Tianjin University of Traditional Chinese Medicine. The patients/participants provided their written informed consent to participate in this study.

## Author Contributions

XZ, YW, and SC participated in the design of the study. YW and SC carried out the acupuncture manipulation and analyzed the data. ZM and RW collected the sample. YW, SC, PP, ZL, and ZZ evaluated the sample of the subjects. HZ and YW wrote the manuscript. All authors contributed to the article and approved the submitted version.

## Conflict of Interest

The authors declare that the research was conducted in the absence of any commercial or financial relationships that could be construed as a potential conflict of interest.

## Publisher’s Note

All claims expressed in this article are solely those of the authors and do not necessarily represent those of their affiliated organizations, or those of the publisher, the editors and the reviewers. Any product that may be evaluated in this article, or claim that may be made by its manufacturer, is not guaranteed or endorsed by the publisher.
